# Artificial Intelligence-Assisted Optimization of *Amanita caesarea* Extracts for Bioactive Compounds and Functional Food Applications

**DOI:** 10.3390/foods15111896

**Published:** 2026-05-28

**Authors:** Mustafa Sevindik, İskender Karaltı, Aras Fahrettin Korkmaz, Tetiana Krupodorova, Ayşenur Gürgen, Ilgaz Akata

**Affiliations:** 1 Department of Biology, Faculty of Engineering and Natural Sciences, Osmaniye Korkut Ata University, Osmaniye 80000, Türkiye; sevindik27@gmail.com; 2 Department of Nutrition and Dietetics, Faculty of Health Sciences, Şirinevler Campus, İstanbul Kültür University, Istanbul 34191, Türkiye; 3 Department of Plant Food Products and Biofortification, Institute of Food Biotechnology and Genomics, National Academy of Sciences of Ukraine, 04123 Kyiv, Ukraine; 4 Department of Industrial Engineering, Faculty of Engineering and Natural Sciences, Osmaniye Korkut Ata University, Osmaniye 80000, Türkiye; aysenurgurgen@osmaniye.edu.tr; 5 Department of Biology, Faculty of Science, Ankara University, Ankara 06000, Türkiye; akata@science.ankara.edu.tr

**Keywords:** edible mushroom, *Amanita caesarea*, functional food, bioactive compounds, antioxidant activity, artificial intelligence optimization, LC-MS/MS

## Abstract

This study evaluated the effects of different extraction optimization approaches on the biological activities and phenolic compositions of the edible mushroom *Amanita caesarea* (Scop.) Pers. Extraction time, extraction temperature and solvent ratio were optimized using Artificial Neural Network–Genetic Algorithm (ANN-GA) and Response Surface Methodology (RSM), while the best experimental extract (BEE) was also included for comparison. The extracts were analyzed for antioxidant parameters (TAS, TOS, OSI, FRAP, and DPPH), antiproliferative and anticholinesterase activities, and phenolic compound profiles by LC-MS/MS. The results showed that the optimization strategy markedly influenced both chemical composition and biological activity. Among the evaluated extracts, the ANN-GA-optimized sample showed the most pronounced biological performance. This extract was characterized by stronger antioxidant activity, a more balanced redox status, enhanced antiproliferative and anticholinesterase effects, and higher amounts of several phenolic constituents, especially gallic acid. Overall, the findings indicate that *A. caesarea* is a promising natural source of bioactive compounds and that AI-assisted optimization can improve its potential use in functional food and nutraceutical applications.

## 1. Introduction

Medicinal and edible mushrooms are considered important natural resources in both nutritional and pharmaceutical terms due to their rich bioactive compounds. These organisms contain various secondary metabolites such as polysaccharides, phenolic compounds, flavonoids, and terpenoids, exhibiting antioxidant, antimicrobial, and antiproliferative effects [1,2]. The extent to which these biological activities can be expressed is closely associated with the extraction conditions applied. Suboptimal extraction parameters may reduce the recovery of bioactive compounds and consequently weaken the observed biological effects. For this reason, optimization strategies based on statistical modeling and artificial intelligence have recently become important tools for improving extraction efficiency and enhancing the reliability of bioactivity-oriented studies [3,4].

*Amanita caesarea *(Scop.) Pers, commonly known as Caesar’s mushroom, is an edible macrofungus species with high nutritional value and is an important organism of scientific interest due to the presence of toxic species within the same genus. Studies on this species mainly focus on morphological description, taxonomic positioning, ecological characteristics, distribution areas, and potential health effects. *A. caesarea* is reported to be characterized by its bright red to orange cap color, distinct striated margin, yellow gills and stem, ring structure, and vesicle-shaped volva at the base [5]. Ecologically, this species is known to form ectomycorrhizal relationships with tree species, especially symbiotic associations with plants belonging to the Fagaceae and Pinaceae families [6]. It is generally found in regions with a hot and dry Mediterranean climate, especially under oak and chestnut trees, and is also observed in pine-oak forests in different geographical areas [5,7]. Geographically, *A. caesarea* is mainly distributed in temperate and Mediterranean regions, particularly in Southern Europe, the Balkans, Anatolia, and parts of Western Asia. Its occurrence is closely associated with ectomycorrhizal host trees, climatic conditions, soil structures, and seasonal precipitation patterns. Therefore, regional ecological differences may influence not only its distribution but also its chemical composition and bioactive compound profile. In terms of edibility, *A. caesarea* is reported to have high gastronomic value and is widely consumed in various countries [5,7,8]. Recent studies have shown that this species is noteworthy not only for its nutritional value but also for its rich bioactive compound content. Extracts obtained from *A. caesarea* are reported to exhibit antioxidant and antimicrobial activities, effectively neutralize free radicals, and contain phenolic compounds such as catechin, epicatechin, cinnamic acid, p-coumaric acid, and ferulic acid [5,9,10]. A direct correlation between the presence of phenolic compounds and antioxidant activity has been suggested, indicating that these compounds enhance the biological activity of the species [11]. Furthermore, the literature contains findings suggesting that *A. caesarea* extracts may exhibit antimicrobial, neuroprotective, and anticholinesterase activity potential. These biological effects are associated with the phenolic compounds and other secondary metabolites contained in the mushroom [9,12,13].

In recent years, artificial intelligence-assisted optimization has become increasingly important in improving extraction efficiency from edible mushrooms and other natural resources. Compared with conventional one-factor-at-a-time methods, artificial intelligence-based models provide a stronger framework for interpreting complex and nonlinear interactions among extraction parameters. These models can also estimate optimum extraction conditions with greater predictive precision. In edible mushroom studies, ANN-based and hybrid optimization approaches have increasingly been applied to improve the extraction efficiency of antioxidant compounds, phenolic constituents, polysaccharides, and other bioactive molecules. These developments are particularly important for functional food and nutraceutical applications, where extraction efficiency, bioactivity, and chemical standardization must be considered together [14,15]. In this context, *A. caesarea* can be considered an important natural resource in terms of both nutritional and biological aspects. Nevertheless, the recovery rate and biological effectiveness of bioactive constituents from this species are strongly influenced by the extraction parameters applied. Therefore, optimizing extraction parameters enables the more efficient recovery of target compounds and contributes to the more accurate evaluation of antioxidant, antiproliferative, and anticholinesterase activities. Statistical and artificial intelligence-based optimization approaches, especially RSM and ANN-GA, offer significant advantages in improving extraction processes [3,16]. These approaches enable the production of extracts with enhanced biological activity and contribute to the more effective evaluation of the biotechnological and pharmacological potential of mushroom-derived natural products. For this purpose, the extraction process of *A. caesarea* was refined through RSM and ANN-GA-based modeling, and the extracts obtained under the selected conditions were examined for their biological response profiles. In this context, antioxidant, antiproliferative, and anticholinesterase activities were evaluated, and phenolic compound profiles were determined to clarify the relationship between extraction strategy, chemical composition, and biological efficacy.

## 2. Materials and Methods

**Figure 1:**
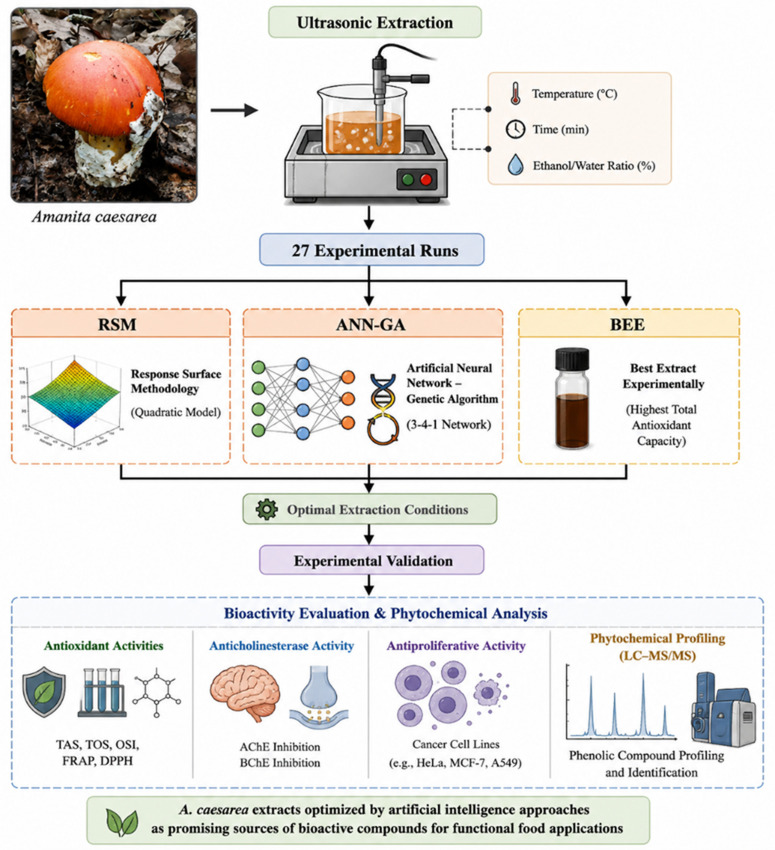
Enlarged schematic representation of the experimental workflow for the optimization and bioactivity evaluation of *Amanita caesarea* extracts.

The macrofungus samples evaluated in the study were obtained from the Belgrad Forest within the borders of Istanbul province, located in the Mediterranean Region of Turkey (Figure 1). The samples collected following the field work were preserved under suitable environmental conditions to prevent deterioration of morphological and taxonomic characteristics and transported to the laboratory as soon as possible. The collected specimens were submitted to the Ankara University fungarium for reference documentation and were registered according to the unit’s routine accession and preservation procedures. The samples were preserved under conditions suitable for fungarium standards for use in subsequent identification and analysis studies.

### 2.1. Extraction Procedure

The extraction experiments were organized using a multivariate experimental design to clarify how the principal process variables jointly influenced extraction performance. In this design, extraction temperature, processing time, and ethanol concentration in the solvent system were considered as the main independent factors. Each factor was examined at three different settings, enabling the design to assess both its independent contribution and its combined behavior with the other variables. Ultrasonic-assisted extraction was conducted under carefully controlled experimental conditions. The extraction time was arranged as 30, 45, and 60 min, while the temperature was adjusted to 30, 45, and 60 °C. In parallel, three solvent compositions were used, corresponding to 0%, 50%, and 100% ethanol in the ethanol/water mixture. The combination of these factor levels resulted in 27 experimental runs. This design provided a systematic basis for determining how extraction duration, temperature, and solvent polarity affected total antioxidant status (TAS) values. Following the extraction experiments, the obtained dataset was initially evaluated by RSM to identify the statistically appropriate range of extraction conditions. In the next stage, ANN-GA modeling was applied to enhance the predictive capability of the optimization procedure and to obtain a more refined estimation of the optimum conditions. The integration of statistical modeling with artificial intelligence-based optimization provided a stronger framework for explaining the nonlinear relationships and mutual interactions among the extraction parameters. Accordingly, the study workflow included ultrasonic-assisted extraction under controlled conditions, followed by optimization using RSM, ANN-GA, and BEE approaches, as shown in Figure 1.

### 2.2. Response Surface Methodology (RSM)

RSM was used to evaluate the effects of extraction temperature, extraction time, and ethanol-water ratio on TAS values. These parameters were defined as independent variables, while TAS was selected as the response variable. Experimental design, statistical analysis, and optimization were performed using Design Expert 13 software. A second-order polynomial equation was used to model the dataset, enabling evaluation of the linear contributions and curvature effects of the extraction factors on TAS responses.

$$Y_{k}=\beta_{k0}+\sum_{i=1}^{n}\beta_{ki}x_{i}+\sum_{i=1}^{n}\beta_{kii}x_{i}^{2}+\sum_{i=1}^{n-1}\sum_{j=i+1}^{n}\beta_{kij}x_{i}x_{j}$$

In the regression equation, the response term Y_k_ was assigned to the TAS values obtained from the extracts. The coded variables X_i_ were used to describe the main extraction factors, namely temperature, extraction time, and solvent composition. The intercept coefficient β_k0_ represented the predicted mean response at the central point of the experimental domain. Model adequacy was assessed using the coefficient of determination (R^2^), analysis of variance (ANOVA), and the corresponding probability values. The optimum extraction conditions were obtained by solving the fitted model equations and identifying the factor levels at which the response reached its maximum value. Three-dimensional response surface graphs were also prepared to illustrate how the independent factors acted together and to clarify the interaction trends among the extraction variables.

### 2.3. Artificial Neural Network–Genetic Algorithm (ANN-GA)

An ANN-based model was developed to improve the prediction of TAS values beyond conventional statistical approaches. Extraction temperature, extraction time, and ethanol-water ratio were used as input variables, while TAS was defined as the output variable. The dataset was divided into training, validation, and test subsets at ratios of 80%, 10%, and 10%, respectively. Network training was performed using the Levenberg-Marquardt algorithm. To determine the optimal network structure, different ANN architectures with 1–17 neurons in the hidden layer were tested and compared. The learning rate and momentum were fixed at 0.5, the maximum iteration number was set to 500, the early stopping limit was 50, and the error tolerance was 1 × 10^−5^.

Each architecture was independently run 1000 times to improve model reliability. Prediction performance was evaluated using mean squared error (MSE) and mean absolute percentage error (MAPE), allowing an objective assessment of the ANN model.

(1) $$MSE=\frac{1}{n}\sum_{i=1}^{n}{\left(e_{i}-p_{i}\right)}^{2}$$

(2) $$MAPE=\frac{1}{n}\sum \left|\frac{e_{i}-p_{i}}{e_{i}}\right|\times 100$$

To assess the predictive capability of the modeling procedure in an objective manner, error-based mathematical indicators were employed. For each experimental run, the measured value was denoted as e_i_, whereas the value estimated by the model was represented as p_i_. The total number of observations was expressed by n, and all error calculations were performed based on this parameter. This evaluation framework made it possible to quantify the differences between experimental observations and model predictions in a systematic way. A Genetic Algorithm (GA) based optimization procedure was then applied to identify the most favorable combination of process variables and to improve prediction accuracy. During optimization, different population sizes were tested, and their influence on convergence behavior and solution stability was comparatively examined. The results indicated that population size played an important role in the efficiency and reliability of the optimization process. New solution alternatives were produced through fitness-based probabilistic selection, and genetic variation in the population was preserved by applying a single-point crossover procedure. This structure supported a broader exploration of the solution space and reduced the possibility of premature convergence to local optimum regions. Throughout the iterative optimization process, the improvement in solution quality was followed using convergence curves, and the relationship between iteration number and optimization performance was evaluated graphically. To strengthen the reliability of the optimization outputs and to support the generalizability of the model, each optimization run was independently repeated 25 times. The results obtained from these repetitions were then statistically analyzed.

### 2.4. Extraction for Bioactivity

To identify the extraction conditions capable of enhancing the biological activity of mushroom extracts, the process was optimized using both statistical and artificial intelligence-assisted multivariate approaches. Based on the RSM results, the optimum extraction conditions were estimated as 49.726 °C, corresponding approximately to 50 °C, 43.915 min, corresponding approximately to 44 min, and an ethanol-water ratio of 54.097%, corresponding approximately to 54% ethanol. The ANN-GA model produced a slightly different optimum profile, predicting 48.8482 °C, approximately 49.0 °C, 38.5908 min, approximately 39 min, and 60.7462% ethanol, approximately 61%, as the most suitable extraction conditions. The optimum conditions predicted by the two models were jointly examined, and validation experiments were carried out using the nearest feasible parameter settings that could be accurately applied in the laboratory. In addition to the model-based optimum extracts, biological activity analyses were also carried out on the extract obtained under the experimental conditions that produced the highest TAS value during the optimization process, defined as the Best Experimental Extract (BEE). To maintain methodological consistency across all applications, the extraction procedures were conducted using an ultrasonic-assisted system. The ultrasonic conditions were kept constant at 40 kHz frequency, 100% power level, and 400 W output power. This standardized procedure improved the comparability of the model-derived results and supported the reliability and reproducibility of the extraction process. After extraction, the ethanol-containing solvent phase was removed under reduced pressure using a rotary evaporator, and the remaining aqueous fraction was completely dried to obtain solvent-free crude extracts. The dried extracts were kept until a constant weight was reached and then reconstituted in DMSO at the required concentrations before bioactivity assays. Thus, possible interference from residual ethanol or water in the biological assays was minimized.

### 2.5. Antioxidant Activity Tests

The antioxidant capacity of the optimized mushroom extracts was determined using several mutually supportive spectrophotometric methods. These assays were selected to evaluate different aspects of antioxidant behavior, including total antioxidant capacity, oxidant level, oxidative balance, radical scavenging ability, and ferric reducing power. Total Antioxidant Status (TAS) was determined using a commercial assay kit (Mega Tıp, Gaziantep, Türkiye). The assay relies on the reduction of the ABTS radical cation (ABTS^•+^) by antioxidant molecules present in the extract. As antioxidants suppress the colored radical form, a decrease in absorbance occurs and is measured spectrophotometrically. TAS values were calculated according to the kit protocol and reported as mmol Trolox equivalent/L [17].

Total Oxidant Status (TOS) was measured to estimate the overall oxidant content of the extracts (Mega Tıp, Gaziantep, Türkiye). In this method, oxidant species in the sample convert ferrous ions into ferric ions. The ferric ions then react with xylenol orange and form a colored complex. The color intensity of the formed complex was measured spectrophotometrically, and TOS values were reported as µmol hydrogen peroxide equivalent/L [18]. OSI was then derived from the TAS and TOS data using the TOS/TAS ratio and presented as a percentage. This index was used to describe the relative balance between oxidant load and antioxidant capacity in the extracts [19].

Free radical scavenging capacity was measured by the DPPH method. For this assay, the extracts were first dissolved in dimethyl sulfoxide to obtain 1 mg/mL stock solutions. Defined volumes of these stocks were then combined with DPPH solution to obtain a uniform reaction mixture. Owing to the photosensitive nature of DPPH, the mixtures were incubated at room temperature under dark conditions until the reaction was complete. The absorbance was recorded at 517 nm, and the results were calculated against Trolox and expressed as mg Trolox equivalent/g extract (mg TE/g) [20].

Ferric reducing capacity was evaluated using the FRAP method. Fresh FRAP reagent was prepared before the assay by mixing acetate buffer (pH 3.6), ferric chloride, and TPTZ solution at appropriate proportions. The extract solutions were combined with the reagent and incubated briefly at 37 °C. During the reaction, ferric ions were reduced to ferrous ions, leading to the formation of the blue ferrous-TPTZ complex. The absorbance of this complex was measured at 593 nm. FRAP activity was calculated from the absorbance values and expressed as mg Trolox equivalent/g extract (mg TE/g) [20].

### 2.6. Anticholinesterase Activity Tests

The anticholinesterase activity of the optimized mushroom extracts was assessed by a spectrophotometric microplate assay adapted from the Ellman method [21]. Acetylcholinesterase (AChE; Type VI-S, EC 3.1.1.7) and butyrylcholinesterase (BChE; EC 3.1.1.8) were selected as target enzymes. Galantamine hydrobromide was included as the standard inhibitor to compare the inhibitory performance of the extracts. For the assay, extract solutions were prepared at different concentrations between 200 and 3125 µg/mL. The reactions were carried out in microplate wells using 0.1 M phosphate buffer at pH 8.0. After the enzyme and extract solutions were added, the mixtures were kept at 25 °C for about 10 min under dark conditions to allow preliminary enzyme–extract interactions. Enzyme activity was started by introducing DTNB together with the corresponding substrate. Acetylcholine iodide served as the substrate for AChE, while butyrylcholine iodide was used for BChE. The hydrolysis reaction led to the formation of a yellow chromogenic product, which was monitored at 412 nm with a microplate reader. Percentage inhibition was derived from the absorbance readings, and the activity of the extracts was reported as IC_50_ values (µg/mL).

### 2.7. Antiproliferative Activity Tests

The antiproliferative activity of the optimized extracts was examined under in vitro conditions using selected human cell lines. A549 lung adenocarcinoma, MCF-7 breast adenocarcinoma, and DU-145 prostate carcinoma cells were used as cancer models, whereas HaCaT human keratinocytes were included as a non-cancerous reference line to assess extract selectivity. All cell lines were supplied by the American Type Culture Collection. To evaluate dose-related effects, extract solutions were prepared at 25, 50, 100, and 200 µg/mL. Cells were maintained in suitable growth media under standard culture conditions. After reaching nearly 70–80% confluence, the cells were harvested with Trypsin-EDTA, transferred into culture plates, and incubated for 24 h to ensure attachment. The culture medium was then replaced with fresh medium containing the extracts at the specified concentrations. After 24 h of exposure, cell viability was determined by the MTT assay. Metabolically active cells converted MTT into formazan crystals, which were dissolved in dimethyl sulfoxide (DMSO). The DMSO-only group served as the negative control. Absorbance values were read at 570 nm and used to compare the responses of cancer cell lines with HaCaT cells [19].

### 2.8. Phenolic Analysis

The phenolic composition of the optimized mushroom extracts was determined using an LC-MS/MS system (Shimadzu LCMS-8030 (Kyoto, Japan)) integrating liquid chromatographic separation with tandem mass spectrometric analysis. This technique enabled the target phenolic and phenolic-like compounds to be detected and quantified in a single analytical run. The standard solution included Alizarin, Thymoquinone, Vanillic acid, Acetohydroxamic acid, Catechin hydrate, Syringic acid, Resveratrol, Gallic acid, Kaempferol, Caffeic acid, Ellagic acid, Silymarin, Chlorogenic acid, Fumaric acid, Protocatechuic acid, Oleuropein, Phloridzin dihydrate, Myricetin, Hydroxycinnamic acid, Naringenin, Quercetin, 2-hydroxy-1,4-naphthoquinone, Luteolin, Salicylic acid, 4-hydroxybenzoic acid, and Curcumin. These reference compounds were selected to cover structurally diverse phenolic groups and to relate the chemical profile of the extracts to their biological responses. Chromatographic separation was performed on a reverse-phase C18 column kept at 40 °C. The mobile phase was composed of two solvents: ultrapure water containing 0.1% formic acid as solvent A and LC-MS-grade methanol containing 0.1% formic acid as solvent B. The system was operated at a flow rate of 0.3 mL/min, and the injection volume was 2 µL. These analytical settings provided adequate separation of the target compounds and allowed their reliable quantification.

### 2.9. Statistical Analysis

Data analysis was performed with IBM SPSS Statistics 21.0 software (IBM Corp., Armonk, NY, USA). Comparisons between two unrelated groups were made using the independent samples *t*-test. When the analysis involved three or more groups, one-way ANOVA was applied to test the presence of significant variation among groups. A significance threshold of *p* < 0.05 was adopted at the 95% confidence level. If ANOVA indicated a significant difference, Duncan’s post hoc test was used to identify the specific group pairs responsible for this difference.

## 3. Results and Discussion

### 3.1. Multivariate Optimization of Extraction Parameters

The extraction factors affecting the TAS of the extracts were examined using a controlled experimental design with multiple factor levels. Temperature was tested at 30, 45, and 60 °C, while extraction time was evaluated at 30, 45, and 60 min. The solvent composition was prepared with ethanol-water ratios of 0%, 50%, and 100%. After extraction under the different combinations of these parameters, TAS values were determined and compared across the experimental groups. The numerical results are given in Table 1.

The effects of extraction temperature, extraction time, and solvent composition on TAS values of mushroom extracts were assessed together. The results showed that antioxidant capacity changed markedly according to both the separate contributions of these factors and their combined interactions. Overall, the 45-min extraction time emerged as the optimum duration yielding the highest TAS values among all temperature and solvent combinations. Specifically, the 5.762 ± 0.039 mmol/L TAS value obtained from extraction at 45 °C and a 50% ethanol/water ratio for 45 min represents the highest antioxidant capacity in the study. The statistically highest value indicates that medium temperature and medium solvent ratio provide the most suitable conditions for the extraction of antioxidant compounds. Similarly, high TAS values (5.684 ± 0.034 and 5.529 ± 0.035 mmol/L) were obtained in systems containing 0% and 100% ethanol at the same time, and these results supported the critical role of the 45-min duration. Similar to the 45 °C conditions, it was determined that the 45-min duration also provided higher TAS values at 30 °C and 60 °C compared to other durations. In this context, the values obtained at 30 °C with 50% and 100% ethanol were measured as 5.632 ± 0.044 and 5.510 ± 0.041 mmol/L, respectively, and at 60 °C as 5.667 ± 0.029 and 5.536 ± 0.031 mmol/L, respectively. These results show that optimizing the extraction time is a decisive factor on antioxidant capacity, independent of temperature and solvent effects. Conversely, when the extraction period was prolonged to 60 min, TAS values declined markedly across all tested temperature levels and solvent compositions. Particularly low TAS values (4.578 ± 0.050–4.670 ± 0.028 mmol/L) obtained at 60 min under high-temperature conditions (60 °C) suggest that prolonged extraction time and high temperature may negatively affect the stability of antioxidant compounds. This indicates that phenolic and similar bioactive compounds are susceptible to thermal degradation and that degradation processes may be activated under prolonged processing conditions. At 30 °C, TAS values displayed a relatively stable pattern across the tested conditions. However, the strongest antioxidant response was obtained at the intermediate temperature level. Under this condition, the highest TAS value was recorded with 50% ethanol after 45 min of extraction. Prolonging the process to 60 min caused a clear reduction in TAS values regardless of the solvent ratio used. In conclusion, it was found that excessive extraction time and temperature limit antioxidant capacity, while a combination of moderate time (45 min), moderate temperature (45 °C), and appropriate solvent ratio (50–100% ethanol) provides the most suitable conditions for obtaining maximum TAS values in mushroom extracts. These findings clearly demonstrate that careful optimization of extraction parameters is critical for the efficient extraction of bioactive compounds.

In our study, two different modeling methods, Response Surface Methodology (RSM) and artificial intelligence-based methods, were comparatively evaluated to improve the efficiency of extraction processes. Within the scope of RSM, four different regression models-linear, two-factor interaction, quadratic, and cubic-were created and analyzed in detail considering basic statistical measures such as coefficient of determination, analysis of variance results, and significance levels. The results showed that the quadratic model provided the best fit for TAS variation, with a coefficient of determination (R^2^) of 0.975. This high level of fit indicates that the model offers a reliable approach for predicting TAS data of *A. caesarea* extracts. Furthermore, the effects of independent variables such as extraction temperature, processing time, and solvent ratio on TAS were found to be statistically significant, indicating that these parameters play a critical role within the model. Consequently, the quadratic regression model emerged as the most suitable approach for statistically improving extraction parameters, both for its high level of prediction accuracy and its ability to facilitate process optimization. The strong fit and reliable predictive capacity offered by the model provide significant advantages, particularly in understanding the interactions between parameters in multivariate systems. The general mathematical expression of the quadratic model is given below:

$$\begin{array}{l} TAS=5.76+0.017\;X_{1}-0.309{\;X}_{2}-0.044\;X_{3}+0.005\;{X_{1}X}_{2}-0.009X_{1}X_{3}+0.026\;X_{2}X_{3}\\ \;\;\;\;\;\;\;\;\;\;\;\;\;\;\;\;\;-0.136\;X_{1}^{2}-0.595{\;X}_{2}^{2}-0.137{\;X}_{3}^{2} \end{array}$$

**Figure 2:**
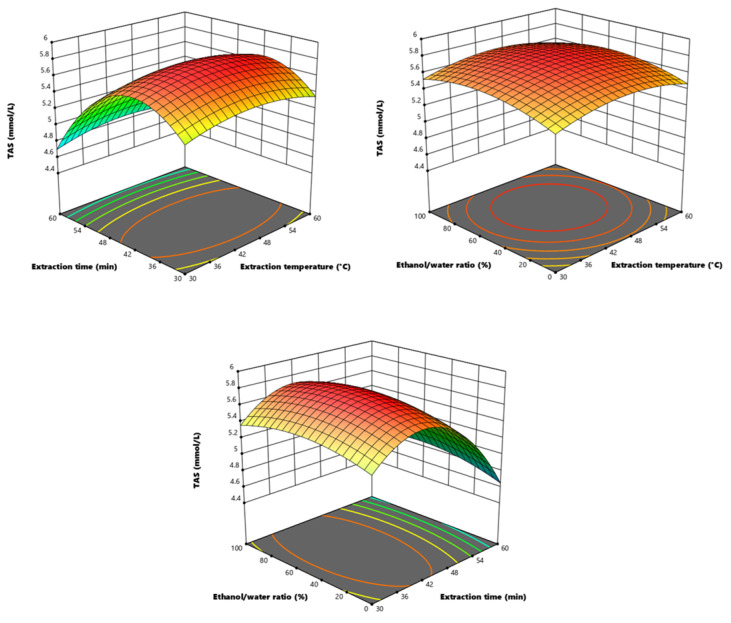
Three-dimensional surface response graphs of extraction parameters.

The second-order regression model developed in this study includes three fundamental variables that define the extraction process. The model included extraction temperature (X^1^), extraction time (X^2^), and ethanol-water composition (X^3^) as independent variables. Their influence on the TAS values of *A. caesarea* extracts was evaluated by considering both individual factor effects and interaction terms. The resulting multivariate responses were presented as three-dimensional surface plots to clarify how these variables shaped the TAS response (Figure 2). These surface plots not only revealed the effects of individual factors but also clearly showed the direction and magnitude of the interactions between the variables. Thus, the synergistic or antagonistic relationships that emerged between the extraction parameters were highlighted, and it was used as an effective visual analysis tool in determining the optimum processing conditions.

In the advanced stage of the modeling process, an artificial neural network-based approach was applied to increase prediction accuracy. Different network architectures were tested, and the structure showing the highest performance was determined to be a 3-6-1 topology consisting of three input, six hidden, and one output neuron. This configuration successfully modeled the nonlinear relationships between extraction parameters and TAS values. The predictive power of the established model was evaluated using three basic statistical measures. The mean square error was calculated as 0.001, indicating that the deviation between the predicted and experimental data was quite low. The mean absolute percentage error was determined as 0.299%, revealing that the model could make predictions with high accuracy. The correlation coefficient reaching a high value of 0.995 clearly shows a strong agreement between the model outputs and experimental results. These findings demonstrate that the artificial neural network model is a reliable and powerful prediction tool with a low error rate and high correlation level. This approach appears to offer significant advantages over classical statistical methods, particularly in the analysis of multivariate and nonlinear systems.

**Figure 3:**
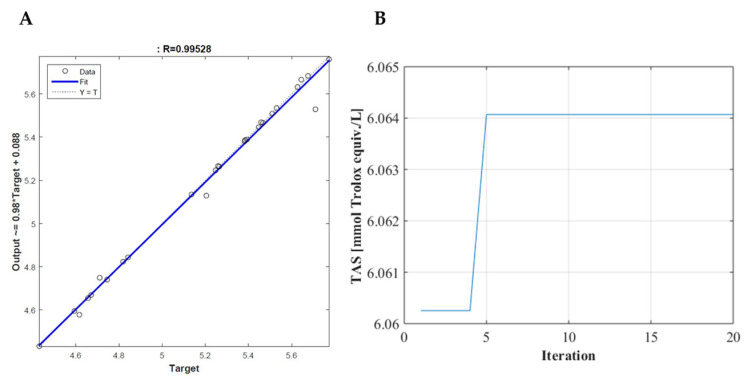
ANN-GA model performance and optimization behavior: (**A**) regression relationship between model predictions and experimental data; (**B**) convergence curve of the genetic algorithm optimization process.

The regression graph presented in Figure 3A reveals a strong linear relationship between model outputs and experimental data (R = 0.99528). The fact that the data points are largely positioned close to the ideal fit line indicates that the model can produce predictions with high accuracy. However, the slope of the regression line being less than 1 (0.98) and the presence of a positive constant term (0.088) indicate that the model shows a tendency to overestimate slightly in low value ranges and underestimate slightly in high value ranges. However, these deviations remained at a very low level and did not significantly affect the overall performance of the model. These results indicate that the model achieved strong predictive performance, with high accuracy, limited error, and good generalization ability.

Using the outputs generated by the artificial neural network, a genetic algorithm-based optimization procedure was then conducted to identify the most suitable extraction conditions. The influence of population size on algorithm performance was tested comparatively. Among the evaluated options, a population size of 10 produced the most consistent and efficient optimization results. During the iterative search process, changes in solution quality were followed using convergence curves. The model reached a stable solution after nearly five iterations and generated values close to the global optimum (Figure 3B). This pattern indicates that the applied optimization strategy provided rapid convergence together with reliable predictive output.

### 3.2. Antioxidant Potentials

Mushrooms with high antioxidant capacity play an important role in suppressing oxidative stress thanks to their chemical structures rich in phenolic compounds, polysaccharides, and various secondary metabolites. These properties make these species prominent natural resources for functional food and pharmaceutical applications [22,23]. The antioxidant performance of the optimized *A. caesarea* extracts was then examined experimentally, and the obtained values are summarized in Table 2.

The present findings indicate that extracts produced through different optimization strategies have distinct effects on redox-related antioxidant parameters. The ANN-GA extract was placed in the statistically highest group for TAS, FRAP, and DPPH values, suggesting a stronger performance in total antioxidant capacity, ferric reducing ability, and free radical scavenging activity. Conversely, the RSM extract showed higher TOS and OSI levels, indicating a relatively greater oxidative burden and a less favorable antioxidant–oxidant balance than the ANN-GA extract. The BEE generally remained at moderate or lower levels across the measured parameters and was mostly included in the lower statistical groups. These results suggest that relying only on the best experimentally observed condition may be insufficient to obtain maximum antioxidant efficiency across multiple parameters.

Various studies in the literature have reported that *A. caesarea* has antioxidant potential using different extraction methods [9,11,12,24,25]. These findings indicate that the species contains considerable levels of phenolic constituents and other bioactive metabolites, which may contribute to its activity mainly through antioxidant-related mechanisms. The findings obtained in the present study are also consistent with this literature data, and show that optimized extraction conditions play a decisive role in antioxidant capacity. However, no previous study has been found regarding the TAS, TOS, and OSI values of *A. caesarea*. Studies on different wild mushroom species have reported TAS values of *Ramaria obtusissima, Hymenopellis radicata, Morchella importuna*, and *Hericium erinaceus* as 6.638, 6.308, 5.266, and 5.426 mmol/L, respectively. In the same studies, TOS values were reported as 10.134, 4.320, 8.144, and 6.621 µmol/L, and OSI values as 0.153, 0.068, 0.155, and 0.122, respectively [26,27,28,29]. While the TAS value reflects the total effect of antioxidant compounds present in a mushroom, the TOS value indicates the level of oxidant compounds. OSI is considered an important parameter indicating the extent to which oxidant compounds are suppressed by the antioxidant system [3].

Compared with previous reports on wild mushroom species [26,27,28,29], the ANN-GA extract in the present study exhibited a competitive TAS value together with lower TOS and OSI levels, indicating a more favorable redox profile. In contrast, the RSM extract was characterized by elevated TOS and OSI levels, indicating a greater oxidative burden, while the BEE displayed a moderate response pattern. These findings indicate that the optimization strategy can influence not only the recovery of antioxidant compounds but also the co-extraction of oxidant components. Therefore, extraction conditions for naturally derived bioactive compounds should be selected according to the targeted biological activity, particularly when extracts are intended for functional food, nutraceutical, or pharmaceutical applications.

### 3.3. Anticholinesterase Activity

Mushrooms with cholinesterase inhibitory activity are considered promising natural sources for modulating neurodegenerative pathways, mainly due to their phenolic constituents and diverse secondary metabolites. These properties increase their relevance for functional food and pharmaceutical applications, particularly in disorders associated with cholinergic dysfunction, such as Alzheimer’s disease [30,31]. Accordingly, the anticholinesterase potential of the optimized *A. caesarea* extracts was tested experimentally, and the results are given in Table 3.

The anticholinesterase activity results obtained in this study showed that the inhibitory effects of *A. caesarea* extracts differed significantly depending on the optimization approach. Since lower IC_50_ values indicate stronger enzyme inhibition, the ANN-GA extract exhibited the highest inhibitory activity against both acetylcholinesterase and butyrylcholinesterase enzymes. This result indicates that ANN-GA-guided extraction created more favorable conditions for recovering compounds linked to cholinesterase inhibitory activity. The RSM extract showed a moderate inhibitory effect, with IC_50_ values higher than those of ANN-GA but lower than those of BEE. In contrast, the BEE had the highest IC_50_ values for both enzymes, indicating the weakest inhibitory activity. This result shows that extraction based only on the best experimental TAS value may not be sufficient to maximize enzyme inhibitory activity, because antioxidant capacity and anticholinesterase potential may not always increase under the same extraction conditions.

Galantamine, used as a standard inhibitor, showed the expected strong inhibitory activity with very low IC_50_ values for both enzymes. However, the higher IC_50_ values of mushroom extracts should be interpreted considering their complex natural composition. Unlike pure pharmaceutical inhibitors, mushroom extracts contain multiple bioactive compounds that may act through weaker but potentially complementary or synergistic mechanisms. Therefore, although the extracts were less potent than galantamine, their activity indicates that *A. caesarea* may contain secondary metabolites capable of interacting with cholinesterase-related pathways.

Previous studies have reported that *Amanita crocea* exhibits inhibitory activity against acetylcholinesterase and butyrylcholinesterase enzymes [32]. This finding supports the view that some species within the genus *Amanita* may contain compounds with cholinesterase inhibitory potential. Compared with these previous data, the present study expands the biological activity profile of edible *Amanita* species by showing that *A. caesarea* extracts also exhibit measurable anticholinesterase effects. In addition, the lower IC_50_ values obtained from the ANN-GA extract compared with RSM and BEE indicate that extraction strategy directly influences the recovery of compounds associated with enzyme inhibition. Overall, these results suggest that AI-assisted optimization may be useful for obtaining mushroom extracts with enhanced cholinesterase inhibitory activity and potential value for functional food, nutraceutical, and neuroprotective applications.

### 3.4. Antiproliferative Activity

**Figure 4:**
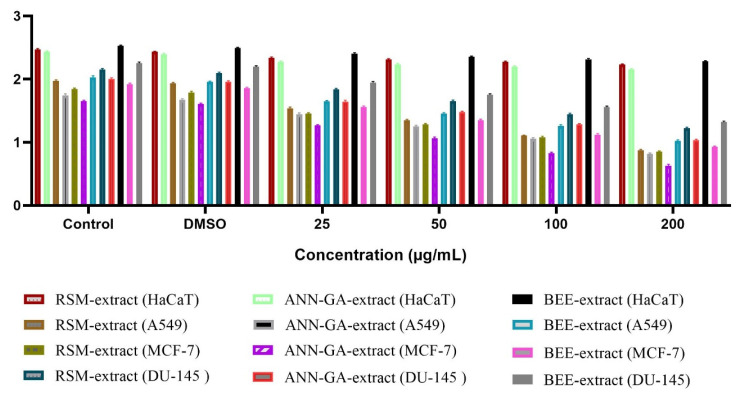
Dose-dependent antiproliferative effects of RSM, ANN-GA, and BEE-optimized *Amanita caesarea* extracts on different human cell lines. Note: Untreated cells grown in standard culture medium were used as the control group, whereas DMSO-treated cells served as the solvent control. The cells were exposed to RSM-, ANN-GA-, and BEE-derived optimized extracts at 25, 50, 100, and 200 µg/mL. The antiproliferative response was examined in HaCaT normal human keratinocytes and in A549, MCF-7, and DU-145 cancer cell lines to determine both concentration-dependent effects and the selectivity profile of the extracts.

Mushrooms with antiproliferative activity are regarded as promising natural sources for cancer-related studies, as their phenolic constituents and diverse secondary metabolites may restrict abnormal cell growth. Owing to these biochemical features, such species are of interest for anticancer drug discovery and pharmaceutical applications [33,34]. Therefore, the antiproliferative responses of optimized *A. caesarea* extracts were assessed experimentally, and the results are shown in Figure 4.

The findings of this study demonstrate that *A. caesarea* extracts exerted a significant and concentration-dependent suppressive effect on cell viability. The decrease in absorbance values with increasing extract concentrations indicates that the antiproliferative effect became more pronounced at higher doses. This dose-dependent trend also suggests that the biological response of the cells was directly influenced by both extract concentration and extraction strategy. When the optimization approaches were compared, the ANN-GA extract caused lower viability values in all tested cell lines and therefore exhibited a stronger antiproliferative effect. In contrast, RSM extracts showed a moderate effect, whereas BEE were generally associated with higher viability values. These findings indicate that artificial intelligence-assisted optimization may improve not only the extraction efficiency of bioactive compounds but also their biological effectiveness.

When evaluated according to cell lines, MCF-7 and A549 cells exhibited a more sensitive profile to the extracts, whereas the DU-145 cell line appeared relatively more resistant. This variation may be associated with cell-specific biochemical and physiological characteristics, such as metabolic activity, antioxidant defense capacity, membrane permeability, and differences in proliferation rate [35]. The stronger response observed in MCF-7 and A549 cells suggests that these cell lines may be more susceptible to the phenolic-rich composition of the optimized extracts. In contrast, the relatively lower response in DU-145 cells may indicate a more resistant cellular phenotype or different sensitivity to mushroom-derived bioactive compounds. Furthermore, the higher viability levels observed in HaCaT cells, which were used as a healthy non-cancerous model, suggest that the extracts may show a certain degree of selectivity toward cancer cells. This selectivity is important because a potential bioactive extract should ideally suppress cancer cell proliferation while causing limited cytotoxicity in normal cells [36].

The results obtained are generally consistent with previous reports on the biological activity potential of *A. caesarea*. Previous studies have shown that methanol extracts of this species exert cytotoxic effects on different cancer cell lines, including MCF-7, HCT-15, CaOV3, PC3, and HeLa cells [25]. Similarly, polysaccharide fractions obtained from *A. caesarea* have been reported to reduce oxidative stress, suppress apoptosis, and prevent mitochondrial damage in HT22 cells, mainly through antioxidant-related mechanisms [12]. These findings suggest that both phenolic compounds and polysaccharide fractions of *A. caesarea* may contribute to its cellular bioactivity through different but complementary mechanisms. In this respect, the present findings support previous evidence showing that *A. caesarea* is not only nutritionally valuable but also biologically active at the cellular level. However, the present study differs from previous studies in several important aspects. Most earlier studies evaluated either a single extraction method or a specific bioactive fraction, whereas this study comparatively examined extracts obtained through RSM, ANN-GA, and BEE strategies. This design made it possible to evaluate how different optimization approaches affect not only chemical composition but also antiproliferative activity. Moreover, the inclusion of HaCaT cells as a healthy cell model strengthened the biomedical interpretation of the results by providing preliminary information on extract selectivity and cytotoxic safety. Therefore, the study provides a more integrated perspective by linking extraction strategy, phenolic composition, antioxidant potential, and cancer cell viability responses.

A standard chemotherapeutic agent was not included as a positive control because the primary aim of this study was to compare the effects of different extraction optimization strategies rather than to evaluate the extracts against clinically used anticancer drugs. However, the HaCaT human keratinocyte cell line was included as a non-cancerous model to provide a preliminary assessment of extract selectivity and cytotoxic safety. The lack of a chemotherapeutic positive control is acknowledged as a limitation and should be addressed in future studies. Overall, these findings indicate that AI-assisted optimization may be a useful approach for obtaining mushroom extracts with improved antiproliferative potential and may support the future evaluation of *A. caesarea* extracts in functional food, nutraceutical, and anticancer-oriented studies.

### 3.5. Phenolic Contents

Phenolic constituents are among the key chemical groups that contribute substantially to the biological activity of fungi. Their presence is closely associated with antioxidant potential and various pharmacological properties [37]. Therefore, the phenolic composition of optimized *A. caesarea* extracts was analyzed comprehensively, and the obtained data are provided in Table 4.

The phenolic compound profile obtained in this study showed that different optimization approaches had a marked effect on the chemical composition of *A. caesarea* extracts. Gallic acid was the most abundant compound detected among all extracts, and its concentration was particularly high in the ANN-GA extract. The strong antioxidant, anticancer, and neuroprotective effects of gallic acid have been widely reported in the literature [36], suggesting that its high level may contribute substantially to the biological activity of the optimized extract. In addition to gallic acid, the presence of caffeic acid, quercetin, and catechin hydrate at relatively high levels may further enhance the antioxidant and cellular bioactivity of the extracts by increasing their free radical scavenging capacity [38,39,40,41]. Thus, both the total phenolic content and compound diversity may contribute to biological activity through possible synergistic effects.

In contrast, the RSM extract showed moderate levels of most phenolic compounds but exhibited the highest value for syringic acid. This indicates that different optimization approaches can create selective effects in the extraction of specific compounds and that extraction parameters may produce compound-dependent responses. In other words, while ANN-GA improved the overall recovery of most phenolic compounds, RSM appeared to provide more favorable conditions for syringic acid extraction. This difference may be related to the compound-specific extraction behavior of syringic acid, which may have responded more effectively to the solvent ratio, temperature, and extraction time predicted by the RSM model. Therefore, this finding indicates that each phenolic compound may respond differently to extraction variables and that a single optimization approach may not maximize all compounds equally.

When the BEE was examined, it generally showed the lowest values for most phenolic compounds and was statistically placed in the lower groups. This result suggests that extraction conditions selected only according to the best experimental TAS value may not be sufficient to maximize the total phenolic yield. Therefore, optimization based on a single response parameter may provide limited efficiency when the target is a complex chemical profile containing multiple phenolic compounds.

Previous studies on the phenolic compound profile of *A. caesarea* reported the presence of compounds such as catechin, cinnamic acid, ferulic acid, and p-coumaric acid, whereas quercetin, gallic acid, and caffeic acid were not detected [9]. The phenolic profile determined in the present study therefore differs from earlier reports. This variation may result from differences in the extraction procedure, solvent composition, analytical method, ecological source of the sample, and, most importantly, the optimization approach applied. In this respect, the RSM and ANN-GA-based extraction approaches applied in the present study may have enabled the recovery of a broader spectrum of phenolic compounds compared with conventional extraction methods.

Phenolic compounds are among the main components responsible for the antioxidant and biological activities of mushrooms [42,43,44]. Therefore, the high antioxidant values obtained in Table 2 are likely associated with the phenolic composition presented in Table 4. In particular, the coexistence of high TAS, FRAP, and DPPH values with high phenolic compound levels in the ANN-GA extract supports the view that phenolics are key contributors to antioxidant activity. The low LOD and LOQ values for all compounds also indicate that the LC-MS/MS method had high sensitivity and reliability. Taken together, these results indicate that AI-guided optimization may enhance the extraction efficiency of phenolic constituents from *A. caesarea*. This approach may also support the use of this mushroom as a source of bioactive compounds for functional food, nutraceutical, and pharmaceutical product development.

## 4. Conclusions

This study demonstrated that extraction parameters and optimization approaches play a decisive role in the chemical composition and biological activities of *A. caesarea* extracts. The findings showed that the extraction strategy affected not only phenolic compound recovery but also antioxidant, antiproliferative, and anticholinesterase activities. Among the tested extracts, the ANN-GA optimized extract showed the most favorable antioxidant profile, with higher TAS, FRAP, and DPPH values and a more balanced redox status. This extract also exhibited stronger antiproliferative and enzyme inhibitory activities, as reflected by lower cell viability values and lower IC_50_ values compared with the other extracts. LC-MS/MS analysis further showed that ANN-GA increased the recovery of most phenolic compounds, particularly gallic acid, whereas RSM was more effective for syringic acid. These differences indicate that phenolic compounds may respond differently to extraction variables and that the most suitable optimization strategy may vary depending on the targeted compound or biological activity.

The primary novelty of this study lies in the side-by-side evaluation of RSM, ANN-GA, and BEE based extraction strategies for *A. caesarea* extracts. Unlike studies generally limited to a single extraction method or a single biological activity, this work evaluated antioxidant redox parameters, antiproliferative activity, anticholinesterase activity, and phenolic composition together. In addition, TAS, TOS, and OSI values were reported for *A. caesarea*, providing a more comprehensive redox-based evaluation of this edible mushroom. Although A. caesarea is a well-documented edible mushroom species, the genus Amanita includes highly toxic taxa. Therefore, the absence of in vivo toxicological evaluation is acknowledged as a limitation, although HaCaT cells were used as a non-cancerous model for preliminary cytotoxic safety assessment. Further studies should focus on in vivo toxicity, bioavailability, isolation of individual phenolic compounds, their synergistic effects, and molecular mechanisms of action. Overall, this study indicates that ANN-GA-based optimization may be an effective approach for improving the functional potential of edible mushroom extracts for food, nutraceutical, and pharmaceutical applications.

## Figures and Tables

**Table 1:** Total antioxidant status (TAS) values of extracts obtained during the optimization process.

Experiment	Solvent/Water (%)	Time (Min)	Temperature (°C)	TAS (mmol/L)
1	0	30	30	5.128 ± 0.055 ^h^
2	0	45	30	5.466 ± 0.031 ^d^
3	0	60	30	4.431 ± 0.048 ^m^
4	50	30	30	5.264 ± 0.034 ^f^
5	50	45	30	5.632 ± 0.044 ^b^
6	50	60	30	4.741 ± 0.041 ^l^
7	100	30	30	5.277 ± 0.032 ^f^
8	100	45	30	5.510 ± 0.041 ^c^
9	100	60	30	4.655 ± 0.042 ^l^
10	0	30	45	5.385 ± 0.055 ^e^
11	0	45	45	5.684 ± 0.034 ^b^
12	0	60	45	4.594 ± 0.035 ^m^
13	50	30	45	5.478 ± 0.031 ^d^
14	50	45	45	5.762 ± 0.039 ^a^
15	50	60	45	4.823 ± 0.030 ^k^
16	100	30	45	5.390 ± 0.032 ^e^
17	100	45	45	5.529 ± 0.035 ^c^
18	100	60	45	4.844 ± 0.047 ^k^
19	0	30	60	5.135 ± 0.033 ^h^
20	0	45	60	5.447 ± 0.037 ^d^
21	0	60	60	4.578 ± 0.050 ^m^
22	50	30	60	5.381 ± 0.021 ^e^
23	50	45	60	5.667 ± 0.029 ^b^
24	50	60	60	4.750 ± 0.037 ^l^
25	100	30	60	5.245 ± 0.017 ^f^
26	100	45	60	5.536 ± 0.031 ^c^
27	100	60	60	4.670 ± 0.028 ^l^

Note: Values indicated by different letters in the same column represent a statistically significant difference (*p* < 0.05, Duncan test).

**Table 2:** Antioxidant profiles of extracts obtained by RSM, ANN-GA, and BEE approaches.

Extracts	TAS (mmol/L)	TOS (µmol/L)	OSI (TOS/(TAS × 10))	FRAP (mg TE/g)	DPPH (mg TE/g)
RSM	5.792 ± 0.022 ^b^	10.032 ± 0.057 ^a^	0.173 ± 0.000 ^a^	252.010 ± 2.420 ^b^	140.920 ± 2.580 ^b^
ANN-GA	6.078 ± 0.032 ^a^	9.072 ± 0.041 ^c^	0.149 ± 0.002 ^c^	265.663 ± 3.810 ^a^	155.350 ± 2.510 ^a^
BEE	5.766 ± 0.004 ^b^	9.377 ± 0.048 ^b^	0.163 ± 0.001 ^b^	245.000 ± 2.590 ^c^	134.753 ± 2.020 ^c^

Note: Values indicated by different letters in the same column represent a statistically significant difference (*p* < 0.05, Duncan test). TAS stands for Total Antioxidant Status; TOS for Total Oxidant Status; OSI for Oxidative Stress Index. FRAP represents ferric reduction antioxidant power; DPPH represents 2,2-diphenyl-1-picrylhydrazyl radical scavenging activity. RSM represents the extract obtained by Response Surface Methodology; ANN-GA represents the extract optimized by Artificial Neural Network and Genetic Algorithm; and BEE represents the extract with the highest experimental TAS value.

**Table 3:** Anticholinesterase activities of RSM, ANN-GA, BEE, and standard inhibitor.

Extracts	AChE (µg/mL)	BChE (µg/mL)
RSM	156.897 ± 1.312 ^b^	212.083 ± 2.323 ^b^
ANN-GA	150.097 ± 1.511 ^c^	203.303 ± 1.783 ^c^
BEE	175.043 ± 2.298 ^a^	221.877 ± 1.578 ^a^
Galantamine	6.477 ± 0.395 ^d^	16.487 ± 0.289 ^d^

Note: Values indicated by different letters in the same column represent a statistically significant difference (*p* < 0.05, Duncan test). AChE represents the acetylcholinesterase enzyme; BChE represents the butyrylcholinesterase enzyme. Galantamine was used as the standard inhibitor. Values are IC50 values.

**Table 4:** Phenolic compounds contents of RSM, ANN-GA, and BEE extracts.

Compounds	LOD	LOQ	RSM Extract	ANN-GA Extract	BEE Extract
Catechin hydrate	7.64	25.47	740.960 ± 1.527 ^b^	1105.750 ± 2.963 ^a^	592.263 ± 1.670 ^c^
Hydroxycinnamic acid	7.79	25.98	1670.830 ± 2.328 ^b^	1888.443 ± 3.243 ^a^	1544.360 ± 1.913 ^c^
4-hydroxybenzoic acid	15.3	51.10	1015.617 ± 5.151 ^b^	1129.453 ± 2.221 ^a^	854.893 ± 1.987 ^c^
Gallic acid	22.88	76.25	9280.923 ± 3.357 ^b^	10,279.223 ± 2.008 ^a^	9131.603 ± 2.641 ^c^
Syringic acid	41.83	139.43	1524.150 ± 2.529 ^a^	1247.610 ± 2.655 ^c^	1465.793 ± 2.802 ^b^
Quercetin	68.40	228.10	525.940 ± 1.583 ^b^	614.973 ± 2.362 ^a^	234.283 ± 0.998 ^c^
Caffeic acid	81.80	272.67	2125.687 ± 2.642 ^b^	2850.503 ± 2.460 ^a^	1951.747 ± 2.366 ^c^

Note: Values indicated by different letters on the same row represent a statistically significant difference (*p* < 0.05, Duncan test). LOD (Limit of Detection) represents the lowest detectable concentration; LOQ (Limit of Quantification) represents the lowest concentration that can be reliably quantified.

## Data Availability

The original contributions presented in this study are included in the article. Further inquiries can be directed to the corresponding author.

## Abbreviations

The following abbreviations are used in this manuscript:

ANN-GA	Artificial Neural Network–Genetic Algorithm
DMSO	Dimethyl sulfoxide
RSM	Response Surface Methodology
MTT	(3-[4,5-dimethylthiazol-2-yl]-2,5-diphenyl-tetrazolium bromide)
TAS	Total Antioxidant Status
DTNB	5.5″-dithiobis-(2-nitrobenzoic acid)
OSI	Oxidative stress index
TOS	Total Oxidant Status
